# Characterization of *Salmonella* Isolates from Wastewater Treatment Plant Influents to Estimate Unreported Cases and Infection Sources of Salmonellosis

**DOI:** 10.3390/pathogens9010052

**Published:** 2020-01-10

**Authors:** Keita Yanagimoto, Takaya Yamagami, Kosei Uematsu, Eiji Haramoto

**Affiliations:** 1 Department of Microbiology, Yamanashi Institute of Public Health and Environment, 1-7-31 Fujimi, Kofu, Yamanashi 400-0027, Japan; yanagimoto-amvs@pref.yamanashi.lg.jp (K.Y.); yamagami-yjz@pref.yamanashi.lg.jp (T.Y.); uematsu-syy@pref.yamanashi.lg.jp (K.U.); 2 Environmental and Social System Science Course, University of Yamanashi, 4-3-11 Takeda, Kofu, Yamanashi 400-8511, Japan; 3 Interdisciplinary Center for River Basin Environment, University of Yamanashi, 4-3-11 Takeda, Kofu, Yamanashi 400-8511, Japan

**Keywords:** *Salmonella enterica*, sewage, unreported case, poultry, wastewater treatment plant

## Abstract

*Salmonella enterica* is a major cause of gastroenteritis usually caused by animal-based contaminated foods. Since the current passive surveillance is not sufficient to detect all infections and infection sources, we determined the prevalence of *Salmonella* isolated from sewage influent of wastewater treatment plants (WWTPs) and compared the characteristics of human and food isolates to identify the infection sources. Sewage influent samples were collected monthly from two WWTPs located in the Yamanashi Prefecture, Japan, for three years. Serotypes, antimicrobial resistances, isolation periods, isolated areas, and pulsed-field gel electrophoresis patterns of six isolates belonging to five serotypes were consistent with those of the isolates from patients. Real-time PCR for *Salmonella* indicated that sewage influents reflect cases of patients infected with *Salmonella*, including unreported cases. Serovars Schwarzengrund and Anatum were predominant in sewage, but not in humans, and their characteristics were closely related or identical to those isolated from poultry heart and liver, respectively. These results suggest that sewage influent contains *Salmonella* isolates from humans and that some originated from unreported human cases infected by poultry-associated products. Therefore, it is necessary to take countermeasures against *Salmonella* infection based on the unreported cases, which would be disclosed by analysis of sewage influent.

## 1. Introduction

*Salmonella enterica* is one of the most important enteropathogenic bacteria, causing approximately 94 million infections and 155,000 deaths annually worldwide [1]. There are more than 1500 serovars in the subspecies enterica, and 99% of them are responsible for human and animal infections [2,3]. In Japan, *Salmonella* is the third leading cause of bacterial food poisoning for a number of patients, as per the Ministry of Health, Labor, and Welfare [4]. As the main reservoir is domestic and wild animals, foods of animal origin, such as beef, pork, poultry, and eggs, are primary sources of foodborne illnesses [3,5,6,7,8]. Among these foods, the prevalence of *Salmonella* in poultry meat in Japan is the highest [7,8]. Since consumption of raw poultry and organ meats is becoming a novel diet in Japan [8], it is important to identify infection sources by obtaining detailed information concerning strains isolated from patients and suspicious foods to prevent persistent infections from these foods. However, the current passive surveillance system is inadequate to collect all these isolates and to observe all the cases associated with *Salmonella* or other pathogens [9], because few asymptomatic cases may be reported to public health officials [10].

Thus far, many investigations have been conducted to estimate the incidence of patients with viruses or bacteria by detecting these pathogens from influents of wastewater treatment plants (WWTPs) [11,12,13,14,15,16,17,18,19,20,21,22,23]. While viruses express host specificity, bacteria can grow if adequate conditions (i.e., temperature, nutrients, and time) are satisfied. Thus, sources of bacteria isolated from influents cannot be determined [10], requiring not only the number of pathogens but also the quality of the isolates from influents to provide an accurate estimate of pathogenic bacterial infections.

This study aimed to determine the prevalence of *Salmonella* isolated from two WWTP influents and to compare the characteristics of these isolates with those from humans in the same study areas for pathogen monitoring and infection source consideration.

## 2. Results

### 2.1. Prevalence of Salmonella Isolates from Sewage Influent Samples and Humans

The frequency of *Salmonella* isolation from sewage influent samples was 99% (71/72), and 689 isolates were grouped into 38 serotypes and 10 untypable strains. Of 157 representative strains comprising 73 and 84 isolates from WWTP-A and WWTP-B, respectively, serovar Schwarzengrund was the most common (11%), followed by Anatum (9%) and then Newport (5%), as presented in Table 1. There was no apparent difference observed in serotypes between isolates from WWTP-A and WWTP-B. Consecutive detections of the same serotype were observed in 15 serotypes (40%) in each WWTP, even in conditions where only one isolate was isolated in 14 serotypes. Six isolates of untypable strain O13:z29:-, serotyped as Agoueve or Cubana, were detected in 7 months, including 5 consecutive months, in samples from WWTP-B.

Conversely, isolates from humans within the same sewage sampling period were grouped into 26 serotypes and 4 untypable strains. Of 77 isolates comprising 46 isolates from humans living in area A, 16 isolates from those in area B, and 15 isolates from those in other areas, serovar Agona was the most common (9%), followed by Stanley (8%), and then untypable strain O4:i:- (8%), which was recognized as a monophasic variant of serovar Typhimurium (Table 2).

Comparing the prevalence of the isolates from sewage samples with that of the isolates from humans, 105 (67%) of 157 sewage isolates and 56 (73%) of 77 human isolates belonging to 19 serotypes were shared, and of these serotypes, 13 serotypes, including Agona, Bareilly, Bovismorbificans, Brandenburg, Colindale, Infantis, Litchfield, Mbandaka, Newport, Saintpaul, Schwarzengrund, Stanley, and untypable (O8:-:1,5), were isolated within 1-month of each other (Table 3).

### 2.2. Antimicrobial Susceptibility

Results of antimicrobial susceptibilities of representative strains revealed that 44 (28%) and 27 (35%) isolates from the concurrent sampling of sewage and humans, respectively, were resistant against at least one antimicrobial agent. Isolates from sewage samples were most frequently resistant against tetracycline (TC) (25%), followed by streptomycin (SM) (19%), and then kanamycin (KM) (13%), whereas isolates from humans were most frequently resistant against SM (27%), followed by TC (25%), and then ampicillin (ABPC) (16%). Resistance against third-generation cephems (ceftazidime (CAZ) and/or cefotaxime (CTX)) was observed for three (Blockley) and one (Infantis) isolate(s) from sewage and humans, respectively, whereas no resistance was observed against carbapenems (imipenem (IPM) and meropenem (MEPM)), fluoroquinolones (ciprofloxacin (CPFX) and norfloxacin (NFLX)), and colistin (CL). In both sources, serovars Agona, Infantis, and Schwarzengrund exhibited a high rate of resistance, ranging from 57% to 100%, whereas serovars Stanley, Saintpaul, Bareilly, and Newport exhibited a lower rate of resistance (0%–40%) (Table 2). The most frequently observed antimicrobial resistance pattern of serovar Agona was SM and TC resistance; serovar Schwarzengrund was resistant against KM, SM, sulfamethoxazole/trimethoprim (ST), and TC, whereas serovar Infantis exhibited variable antimicrobial patterns.

### 2.3. Pulsed-Field Gel Electrophoresis (PFGE) Analysis

**Figure 1:**
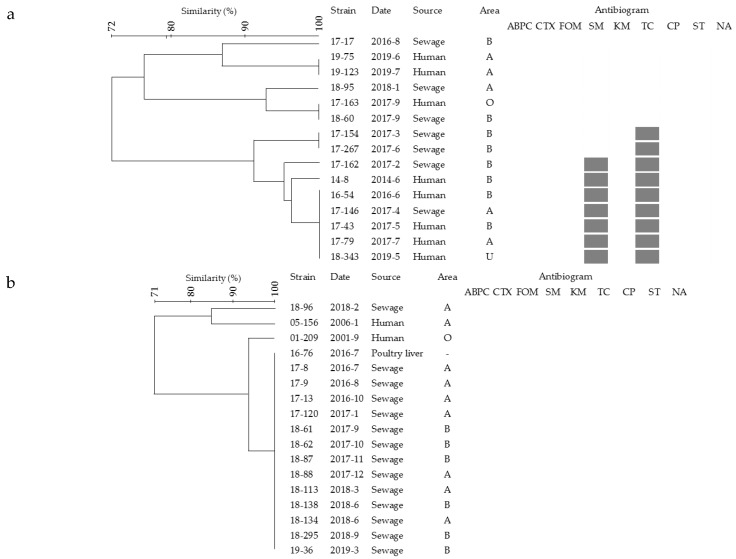

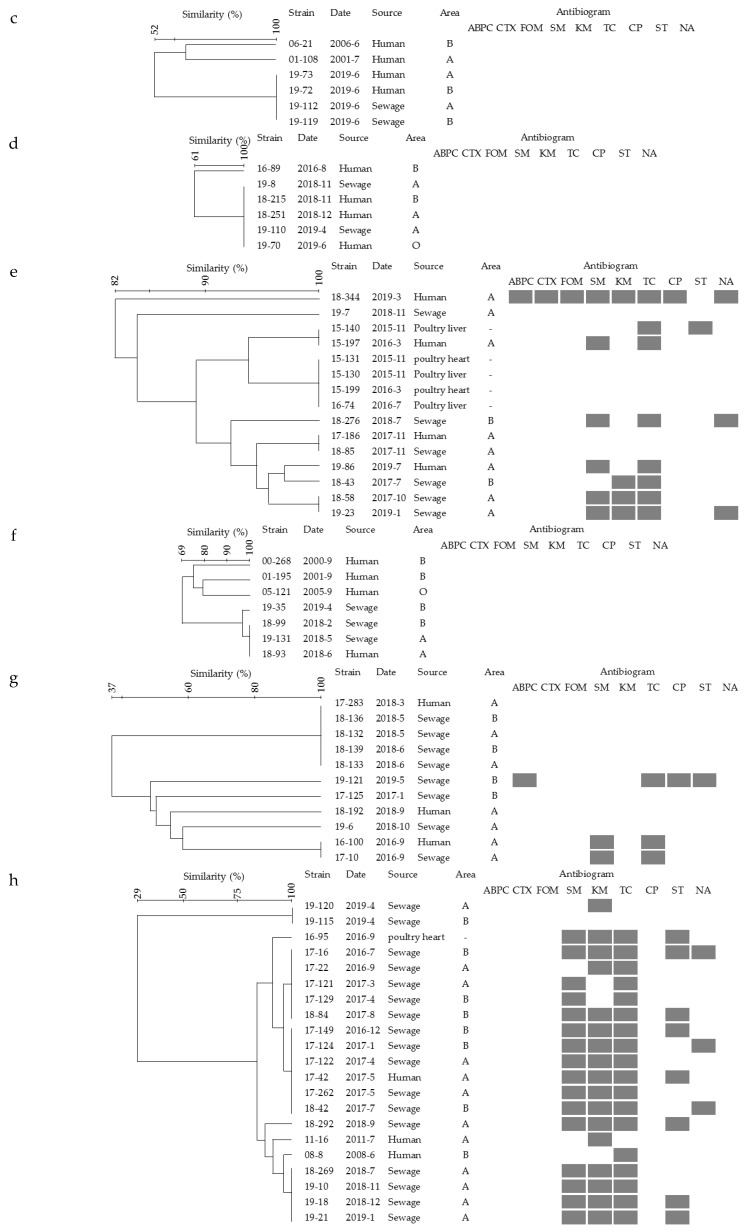
Dendrograms of pulsed-field gel electrophoresis (PFGE) patterns digested with XbaI. Area A and area B, where WWTP-A or WWTP-B is located, respectively; area O, areas other than areas A and B; area U, unknown area. Black squares show resistance against antimicrobial agents. (**a**), Agona; (**b**), Anatum; (**c**), Bovismorbificans; (**d**), Brandenburg; (**e**), Infantis; (**f**), Mbandaka; (**g**), Newport; (**h**), Schwarzengrund.

PFGE analysis of representative strains of the 13 shared serotypes among humans, isolated within a 1-month period, revealed 12 PFGE patterns, belonging to serovars Agona, Bareilly, Bovismorbificans, Brandenburg, Infantis, Mbandaka, Newport, Stanley, and Schwarzengrund; among the same serotypes, isolates from sewage were indistinguishable from those from humans, but strains isolated in the other periods (2000–2011) were not identical to any isolates. Additionally, among these isolates, two each of Agona and Bovismorbificans and one each of Brandenburg, Infantis, Mbandaka, and Newport were isolated within a 1-month period from those from humans (Figure 1a, c–g); furthermore, the isolated area and antimicrobial resistance pattern of two Bovismorbificans and one each of Brandenburg, Infantis, Mbandaka, and Newport were in agreement with those from humans. In serovar Colindale, the PFGE patterns of two isolates from sewage were indistinguishable from each other. Although isolates did not have identical PFGE patterns, the pattern difference between isolates from sewage and humans was only two bands, and their similarity was 90%.

To estimate the sources of the isolates from sewage, the serovar Agona strain, designated as 14-8, which was isolated from a human in 2014 and clustered with strains isolated from broilers using PFGE analysis and whole-genome sequencing [24,25], was also analyzed. The PFGE pattern of the 14-8 strain was closely related to those of two and four isolates from sewage and humans, respectively, and the antimicrobial resistance pattern was consistent with these six isolates (Figure 1a). Additionally, the PFGE pattern of strain 16-95 isolated from retail poultry heart and belonging to serovar Schwarzengrund, which was the most frequent serotype isolated from sewage, showed high similarity to PFGE patterns of 10 isolates from sewage and one from human, and the antimicrobial resistance pattern was consistent with two isolates from sewage and one isolate from human (Figure 1h). Moreover, the PFGE pattern of the 16-76 strain, isolated from retail poultry liver and belonging to serovar Anatum, which was the second frequent serotype isolated from sewage, was identical to those of 13 isolates from sewage, and no antimicrobial resistance was observed among these isolates (Figure 1b). 

### 2.4. Quantification of Salmonella from Sewage Influent Samples

**Figure 2:**
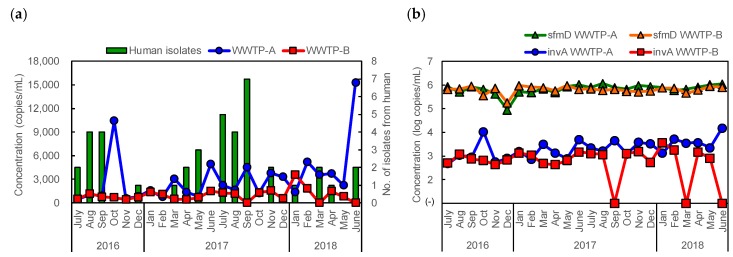
Concentration of *invA* in sewage influent from WWTP-A and WWTP-B determined by real-time PCR and the number of isolates from humans in 2 years (**a**). Concentration of *invA* and *sfmD* in sewage from WWTP-A and WWTP-B (**b**).

The *Salmonella*-specific gene (*invA*) was detected by real-time PCR in all but three WWTP-B samples. The average *invA* concentration of samples from WWTP-A was significantly higher than that from WWTP-B samples (*p* < 0.01). Correlations between *invA* concentration and the number of human isolates in each month were not significant (*p* = 0.83 in WWTP-A, 0.62 in WWTP-B) (Figure 2a). Although the seasonal trend of *invA* concentration was not observed, fluctuation in *invA* concentration was significantly observed in a comparison with that of *sfmD* (*p* < 0.01), which is a specific gene of *Escherichia coli* (Figure 2b).

## 3. Discussion

It is important to compare the characteristics of isolates derived from the same region since there are differences in the reported serotypes among the continents [3,26], or even within Japan. For example, the most frequent serotypes isolated from humans were untypable strain O4:i:-, followed by Infantis, Manhattan, and Typhimurium, in the Hokkaido Prefecture (northern area) in 2014–2015 [27], whereas Thompson, followed by Corvallis, and then Schwarzengrund, was the most prevalent in the Miyazaki Prefecture (southern area) in 2013–2014 [28].

In this study, 19 serotypes of *Salmonella* isolated from sewage from WWTP-A or WWTP-B were also isolated from humans living in the region or visiting hospitals where WWTP-A or WWTP-B was located; antimicrobial resistance, isolation periods, isolated area, and PFGE patterns of six isolates belonging to five serotypes were consistent with those from humans (Figure 1c–g). This result suggests that isolates from sewage are strongly related to those from humans.

Serovar Colindale, which was rarely isolated from humans in Japan [29] but was isolated from enteric infection cases < 5 years in Gambia [30] and lettuce in Burkina Faso [31], had never been isolated from humans from 1985 to 2017 in the Yamanashi Prefecture, mainly area A and area B, in previous studies on more than 3200 *Salmonella* strains in total [32,33] (unpublished data). However, we concurrently isolated this serotype in August 2018 from two isolates of sewage and human origins, and the PFGE pattern of these isolates showed a high similarity (Table 3). Besides, serovar Bovismorbificans, which caused outbreaks from contaminated fresh sprouts and ham products in Germany [34] and the Netherlands [35], respectively, was rarely identified from humans in Japan [29], and only five strains, out of more than 3300 strains, were isolated from humans in the Yamanashi Prefecture between 1985 and 2018 [32,33] (unpublished data). Here, all isolates from two each of sewage and human samples, belonging to this serotype, were concurrently identified in June 2019 (Table 3), and their PFGE patterns were indistinguishable from each other (Figure 1c). Moreover, serovar Agona strain 14-8, which was isolated from human and clustered with the isolates from broilers in the Yamanashi Prefecture using whole-genome sequencing [24], was closely related to the isolates from two sewage and four human samples (Figure 1a). Murakami et al. [36] and Torii et al. [25] pointed out that the strains clustered with strain 14-8 were rarely obtained from broilers, other retail poultry, or humans in other regions. Furthermore, serovar Mbandaka, which caused an outbreak linked to cereal in the United States between March and August 2018 [37,38], was detected from sewage and humans in May and June 2018, respectively, although the relationship to the outbreak strain was not accounted for. This serotype is also hardly isolated in the Yamanashi Prefecture [32,33] (unpublished data), and isolate characteristics were found to be consistent (Figure 1f). It seems unlikely that these non-usual serotypes from human and geographically distinctive strains would be detected in the same period coincidentally if there was no correlation between the isolates from sewage and those from humans.

Here, consecutive detections of isolates belonging to the same serotype appeared from months 2 to 5 (Table 1). We reason that consecutive detection is mainly due to human sources based on the following three reasons: First, the excretion periods of this pathogen from humans would be longer than 1 month even if the administration of antimicrobial agents were conducted [39]. Second, the amount of *Salmonella* discharged from other sources, such as contaminated foods, including poultry, which is the most contaminated in Japan, is estimated to be much lower (<10 is the most probable number/g) [40] than that from patients (10^5^–10^7^ colony-forming units/g) [41]. Third, according to Diemert et al. [16], it is unlikely that persistent detections occurred due to the formation of biofilms inside sewage pipes for months.

Real-time PCR for *Salmonella* showed that the *invA* concentration in sewage at WWTP-A was significantly higher than that at WWTP-B and that fluctuation of concentration was significantly higher in *invA* than in *sfmD* as an indicator of human fecal concentration (Figure 2b). A higher concentration of *invA* at WWTP-A was consistent with more isolates from humans in area A, and the fluctuation of concentration could be interpreted as a *Salmonella* outbreak in the community. These results suggest that the concentration of *invA* in sewage reflects the number of humans infected with *Salmonella*, including unreported cases. It is impossible to estimate the number of humans infected with *Salmonella* because of non-human sources in sewage [42]; however, a considerable number of unreported human cases seems to contribute to the *invA* concentration given the lack of correlation between *invA* concentration and the number of reported human cases. Our result that no seasonal trend was observed conflicts with those reported by Kacprzak et al. [20] and Yan et al. [15], who reported the concentration of *Salmonella* to be highest in autumn and summer in Poland and Hawaii, respectively; this could be due to food culture, geographical circumstance, and hygiene conditions. In Yamanashi, Japan, unreported cases of Salmonellosis were detectable in all seasons.

Concurrent detection of rare serotypes or isolation of rare strains in sewage and human, consecutive detections of the same serotype, and quantification of *invA* indicate that sewage contains *Salmonella* isolates from humans and has the ability to provide an estimate of the epidemic. These results are consistent with the results reported by Yan et al., who demonstrated that *Salmonella* isolates from municipal wastewater were identical to the clinical isolates in terms of isolation periods and PFGE patterns in Hawaii [15]. However, there are gaps in the serotype of isolates between sewage and humans. For example, serovars Schwarzengrund and Anatum were predominant among the isolates from sewage; however, of these serotypes, only one isolate of Schwarzengrund was identified from human (Table 1 and Table 2). It is possible to consider that these isolates from sewage originated from unreported human cases including clinical and non-clinical cases infected by poultry-associated products for the following three reasons. First, Berge et al. stated that more variability in serovars isolated from wastewater suggests that the sources of *Salmonella* isolates from wastewater include both unreported clinical and non-clinical cases [10], and Diemert et al. reported that statistically dominant serotypes in municipal wastewater should be representative of clinical cases or asymptomatic shedding [16]. Here we demonstrated that more kinds of serotypes were detected in sewage, and that Schwarzengrund and Anatum were the predominant serotypes (Table 1). Second, real-time PCR revealed that no seasonal trend was observed, and the concentration of *invA* in sewage did not correlate with that in human isolates (Figure 2a). Third, the PFGE and antimicrobial resistance patterns of serovars Schwarzengrund and Anatum isolated from sewage were closely related or identical to those of these two serotypes isolated from poultry heart and poultry liver retailed in area A, respectively (Figure 1b,h).

Aside from these reasons, there is a unique circumstance in Japan; although there is a law by the Food Sanitation Act prohibiting serving raw meats except for raw poultry including raw organ meats, such as beef without a heating surface, raw beef liver, raw pork, and raw pork organ meats at restaurants, it is reported that the prevalence of *Salmonella* in retail poultry and poultry organ meats was higher than that of beef, pork, and beef organ meats [7,8]. Therefore, the consumption of contaminated raw or undercooked poultry is recognized as a major route of *Salmonella* infection [5]. This means people in Japan have more opportunities to consume more *Salmonella*-contaminated foods, including raw poultry and poultry organ meats. Thus, it is essential to serve poultry and poultry organ meats, which are well heated with considerable care.

Additionally, serovar Agona, which was closely related to the isolate from humans with broiler-related characterization in six isolates, predominated in both sewage and human samples. The prevalence differences in humans between serovar Agona and serovars Anatum and Schwarzengrund suggest that serovar Agona possesses higher pathogenicity. According to the Ministry of Health, Labor, and Welfare [3], no contaminated poultry, or poultry organ meats were responsible for *Salmonella*-associated foodborne outbreak in 2016–2018 in Japan, but some infections were suggested in our study. These results indicate that the actual number of infected humans with relatively mild pathogenicity serotypes, which may include serovars Anatum and Schwarzengrund, is much higher than expected from the current passive surveillance system.

Reilly et al. [17] explained that asymptomatic cases may result from adequate immunity in the hosts or the lack of contamination degree, which is present in infection sources. There is a possibility that an increase in the number of asymptomatic cases causes a proportional increase in the number of symptomatic cases. Additionally, the isolates showing identical characteristics, such as serovars Anatum (18-134, 18-138) (Figure 1b), Bovismorbificans (19-112, 19-119) (Figure 1c), Colindale, and Newport (18-132, 18-136) (Figure 1g), were detected in the same month from sewage in both WWTP-A and WWTP-B; this indicates that the number of humans infected with these strains was more than one and that the infection widely occurred by the same source at the same time, which may be referred to as an outbreak, even though there was no outbreak reported for the duration. Therefore, it is inadequate to take countermeasures against *Salmonella* infections by considering only the reported cases. Although it is impossible to identify these infection sources clearly, foods including poultry-associated products are one of the most suspicious sources.

Limitations of our study are the relatively low frequency of sewage sampling and the small number of isolates from foods. Further investigation for comparing the characteristics of isolates from sewage with isolates from suspicious infection sources should be conducted to understand actual infections and to take an appropriate countermeasure against hidden infection sources for preventing *Salmonella* infections.

## 4. Materials and Methods

### 4.1. Sample Collection

Sewage influent samples were collected monthly from WWTP-A and WWTP-B in the Yamanashi Prefecture, Japan, for 3 years between July 2016 and June 2019. These WWTPs serve a population of ~350,000 (corresponding to ~40% of the population in the Yamanashi Prefecture) and treat ~180,000 m^3^/day of waste in total. All samples were collected under normal weather conditions, stored at 4 °C after sampling, and tested within 24 h.

### 4.2. Isolation of Salmonella

Four hundred milliliters of each sample was concentrated by centrifugation at 21,000× *g* for 25 min to prepare 2 mL suspensions; 0.1 mL of each suspension was added to 10 mL of Rappaport–Vassiliadis broths (Eiken Chemical, Tokyo, Japan) and incubated at 42 °C for 20–24 h. The culture was inoculated onto CHROMagar *Salmonella* (CHROMagar, Paris, France) and *Salmonella*–*Shigella* agar plates (Eiken Chemical) and incubated at 35 °C. Ten suspected colonies were isolated and confirmed as *Salmonella* species by biochemical examinations using triple sugar iron medium and lysine–indole-motility medium; the isolates were serotyped using a commercial *Salmonella* O and H antiserum set (Denka Seiken, Tokyo, Japan).

### 4.3. Strains

Representative *Salmonella* strains isolated from each sample were selected based on their serotypes and biochemical characteristics. Then, 93 isolates from humans mainly living in the areas where WWTP-A and WWTP-B were located, designated as area A and area B, respectively, which were provided by hospitals and inspection institutes between September 2000 and July 2019 and serotyped as described above, and seven isolates obtained between November 2015 and September 2016 from poultry organ meats retailed in area A in a previous report [43] were analyzed for comparison. No outbreak strain of *Salmonella* was included.

### 4.4. Antimicrobial Susceptibility Test

Representative strains were tested for their antimicrobial susceptibility patterns based on the Kirby–Bauer disc diffusion method using BBL Sensi-Disc susceptibility test discs (BD, Tokyo, Japan) on Mueller–Hinton II agar (BD). The results of the following antimicrobials against the isolates were interpreted in accordance with the Clinical and Laboratory Standards Institute criteria [44]: ABPC, cefoxitin, CTX, CAZ, IPM, MEPM (β-lactams), fosfomycin (fosfomycin), CL (polypeptides), SM, KM, amikacin, gentamicin (aminoglycosides), TC (tetracyclines), chloramphenicol (phenicols), ST (sulfonamides and trimethoprim), nalidixic acid, NFLX, and CPFX (quinolones). *E. coli* ATCC25922 was used as the quality control strain.

### 4.5. PFGE Analysis

To evaluate the similarities between the representative strains, PFGE was performed as described previously by Ribot et al. [45] with some modifications. Briefly, cell suspensions were prepared from plates directly, and agarose plugs were solidified using 1% gold agarose (Seakem, Cambrex, NJ) and treated with 1 mg/mL proteinase K solution with 1% N-lauroylsarcosine. Bacterial DNA was digested with 30 U of XbaI (Takara Bio, Kusatsu, Japan) for 4 h at 37 °C, and PFGE was conducted using the CHEF Mapper apparatus (Bio-Rad, Tokyo, Japan). The PFGE patterns were analyzed with Quantity One (Bio-Rad), and dendrograms were constructed based on the unweighted pair group method using arithmetic average algorism.

### 4.6. Quantification of Salmonella

Bacterial DNA was extracted from 250 μL of concentrated samples collected between July 2016 and June 2018 using the QIAamp PowerFecal DNA Kit (Qiagen, Tokyo, Japan) as per the manufacturer’s protocol. The quantification of bacterial DNA was performed by real-time PCR using the Thermal Cycler Dice Real Time System TP 800 (Takara Bio). The amplification of *invA* and *sfmD* was carried out with primer pairs as described previously by Iijima et al. [46] and Kacilikova et al. [47], respectively. Each 25 μL reaction mixture contained 2.5 μL of template DNA, 12.5 μL of 2× probe qPCR mix with UNG (Takara Bio), 0.1 μL each of 100 pmol/μL forward and reverse primers, and 0.05 μL of 100 pmol/μL TaqMan probe. PCR was conducted under the following conditions: initial denaturation at 95 °C for 30 s, followed by 45 cycles at 95 °C for 5 s, and 60 °C for 30 s. A standard curve was obtained using artificially synthesized plasmid DNA containing amplification region sequences of *invA* or *sfmD*, which was diluted serially by tenfold. All samples were tested in duplicate, and the average cycle threshold values were used for calculation. 

### 4.7. Statistical Analysis

Differences between the number of *invA* copies extracted from sewage samples collected from WWTP-A and WWTP-B or the concentration of *invA* and *sfmD* were compared using the t-test and F-test. Tests for homogeneity of variance to compare the fluctuation of *invA* and *sfmD* concentrations were conducted using common logarithms. The correlation between the concentration of *invA* in sewage and the number of human isolates was analyzed using regression analysis. *P* values < 0.01 were considered significant.

## 5. Conclusions

In conclusion, by analyzing the isolates from sewage at two WWTPs, we revealed that the unreported cases, including clinical and non-clinical cases, occur more frequently than expected from the current passive surveillance. Moreover, the expected infection sources contain contaminated poultry organ meats, which are not prohibited to be served for raw consumption at restaurants in Japan. Therefore, it is necessary to take countermeasures against *Salmonella* infection by considering unreported cases, which could be disclosed by the analysis of sewage influent.

## Figures and Tables

**Table 1:** Prevalence of *Salmonella* spp. isolated from sewage influent samples at wastewater treatment plant (WWTP)-A and WWTP-B.

Serotype	2016						2017												2018												2019						Total (%)	
	7	8	9	10	11	12	1	2	3	4	5	6	7	8	9	10	11	12	1	2	3	4	5	6	7	8	9	10	11	12	1	2	3	4	5	6		
Schwarz-engrund	B	-	A	-	-	B	B	-	A	A, B	A	-	B	B	-	-	-	-	-	-	-	-	-	-	A	-	A	-	A	A	A	-	-	A, B	-	-	17	(11)
Anatum	A	A	-	A	-	-	A	-	-	-	-	-	-	-	B	B	B	A	-	A	A	-	-	A, B	-	-	B	-	-	-	-	-	B	-	-	-	14	(9)
Newport	-	A	-	-	-	-	B	-	-	-	-	-	-	-	-	-	-	-	-	-	-	-	A, B	A, B	-	-	-	A	-	-	-	-	-	-	B	-	8	(5)
Saintpaul	-	-	-	-	A	-	B	-	A	-	-	B	-	B	-	A	-	B	-	-	-	-	-	-	-	-	-	-	-	-	-	-	-	-	-	-	7	(5)
Agona	-	B	-	-	-	-	-	B	B	A	-	B	-	-	B	-	-	-	A	-	-	-	-	-	-	-	-	-	-	-	-	-	-	-	-	-	7	(5)
Thompson	-	-	-	-	B	B	-	-	-	-	-	-	-	-	-	-	-	-	-	-	A	-	-	-	-	-	B	-	B	A	-	A	-	-	-	-	7	(5)
Bareilly	-	-	-	A	-	-	-	-	-	-	-	-	-	-	A	-	-	-	-	-	-	A	-	-	A	-	A	B	-	-	-	B	-	-	-	-	7	(5)
Infantis	-	-	-	-	-	-	-	-	-	-	-	-	B	-	-	A	A	-	-	-	-	-	-	-	B	-	-	-	A	-	A	-	-	-	-	-	6	(4)
Braenderup	-	-	-	-	-	-	-	-	-	-	-	-	-	B	-	-	-	-	-	-	-	-	-	-	-	-	-	-	B	B	A, B	-	-	-	-	-	5	(3)
Chester	-	-	-	-	-	B	-	B	B	-	-	A	-	-	-	-	-	-	-	-	-	-	-	-	-	-	-	-	-	-	-	-	-	B	-	-	5	(3)
Stanley	-	-	A	-	-	-	A	-	A	-	-	-	-	A	-	-	-	-	-	-	-	-	-	-	-	-	-	-	-	-	-	-	-	-	-	-	4	(3)
Mikaw-ashima	-	-	-	-	-	-	-	-	-	B	-	-	-	-	-	-	-	-	-	-	-	-	-	-	-	B	B	-	-	-	-	B	-	-	-	-	4	(3)
Corvallis	-	-	-	-	-	-	A	-	A	-	-	-	-	-	-	-	-	-	-	-	-	-	-	-	A	B	-	-	-	-	-	-	-	-	-	-	4	(3)
Hvittingfoss	-	-	B	-	-	-	-	-	-	-	A	-	A	-	-	-	-	-	-	-	-	-	-	-	-	-	-	-	-	-	-	-	-	-	-	-	3	(2)
Blockley	-	-	-	-	-	-	-	-	B	B	B	-	-	-	-	-	-	-	-	-	-	-	-	-	-	-	-	-	-	-	-	-	-	-	-	-	3	(2)
London	-	-	-	B	-	-	-	-	B	-	-	-	-	-	-	-	-	-	-	-	-	B	-	-	-	-	-	-	-	-	-	-	-	-	-	-	3	(2)
Mbandaka	-	-	-	-	-	-	-	-	-	-	-	-	-	-	-	-	-	-	-	B	-	-	A	-	-	-	-	-	-	-	-	-	B	-	-	-	3	( 2)
Cerro	-	-	-	-	-	-	-	-	-	-	-	-	-	A	A	-	-	-	-	-	-	-	-	-	-	A	-	-	-	-	-	-	-	-	-	-	3	( 2)
Altona	-	-	-	-	-	-	-	-	-	-	-	-	-	-	-	-	-	-	-	-	-	-	-	-	-	-	-	-	-	-	-	A	A	-	B	-	3	( 2)
Rissen	-	-	-	-	-	A	-	-	-	-	-	B	-	-	-	-	-	-	-	-	-	-	-	-	-	-	-	-	-	-	-	-	-	-	-	-	2	( 1)
Vitkin	-	-	-	-	-	-	-	-	-	-	-	-	-	-	-	-	-	-	-	-	-	B	B	-	-	-	-	-	-	-	-	-	-	-	-	-	2	( 1)
Colindale	-	-	-	-	-	-	-	-	-	-	-	-	-	-	-	-	-	-	-	-	-	-	-	-	-	A, B	-	-	-	-	-	-	-	-	-	-	2	( 1)
Brande-nburg	-	-	-	-	-	-	-	-	-	-	-	-	-	-	-	-	-	-	-	-	-	-	-	-	-	-	-	-	A	-	-	-	-	A	-	-	2	( 1)
Bovismo-rbificans	-	-	-	-	-	-	-	-	-	-	-	-	-	-	-	-	-	-	-	-	-	-	-	-	-	-	-	-	-	-	-	-	-	-	-	A, B	2	( 1)
Litchfield	-	-	A	-	-	-	-	-	-	-	-	-	-	-	-	-	-	-	-	-	-	-	-	-	-	-	-	-	-	-	-	-	-	-	-	-	1	( 1)
Aberdeen	-	-	-	-	-	-	B	-	-	-	-	-	-	-	-	-	-	-	-	-	-	-	-	-	-	-	-	-	-	-	-	-	-	-	-	-	1	( 1)
Typhim-urium	-	-	-	-	-	-	-	-	-	-	-	-	-	-	-	-	-	-	-	-	-	-	-	-	-	-	-	-	-	-	-	A	-	-	-	-	1	( 1)
Derby	-	-	-	-	-	-	-	-	-	-	-	-	-	-	-	-	-	-	-	-	-	-	-	-	-	-	-	-	-	-	-	B	-	-	-	-	1	( 1)
Ebrie	-	-	-	-	-	-	-	-	-	-	-	-	-	-	-	-	-	-	-	-	-	-	-	-	-	-	-	-	-	-	-	-	-	B	-	-	1	( 1)
Javiana	-	-	-	-	-	-	-	-	-	-	-	-	-	-	-	-	-	-	-	-	-	-	-	-	-	-	-	-	-	-	-	-	-	A	-	-	1	( 1)
Untypable (O13:z29:-)	-	-	-	-	-	-	-	-	-	-	-	-	-	-	-	-	-	-	B	-	B	A, B	B	B	B	-	-	-	-	-	-	-	-	-	-	-	7	( 5)
Untypable (O7:HNM)	-	-	-	-	-	-	-	-	-	-	-	-	-	-	-	-	B	B	-	-	-	-	-	-	-	-	-	-	A	-	-	-	-	-	-	-	3	( 2)
Untypable (O8:-:1,5)	-	-	-	-	-	-	-	-	-	-	-	-	-	B	-	-	-	-	-	-	-	-	-	-	-	-	-	-	-	-	-	-	-	-	-	-	1	( 1)
Others	-	-	A	-	-	-	B	-	-	B	-	A	B	-	-	-	-	B	B	-	A	-	-	B	A	-	-	-	-	A, B	-	B	B	B	A	A	17	(11)
Total	2	3	5	3	2	4	8	2	8	6	3	5	4	6	4	3	3	4	3	2	4	5	5	6	6	5	5	2	6	5	4	7	4	7	3	3	157	

A shows isolation from WWTP-A; B shows isolation from WWTP-B. Total is not 100% due to the approximation of values.

**Table 2:** Antimicrobial resistance of the isolates from sewage influents and humans.

Serotype	Sources							
	Sewage				Human			
	No. of Isolates (%)		No. of Antimicrobial-Resistant Isolates (Rate of Resistance; %)		No. of Isolates (%)		No. of Antimicrobial-Resistant Isolates (Rate of Resistance; %)	
Agona	7	(5)	4	(57)	7	(9)	4	(57)
Stanley	4	(3)	0		6	(8)	0	
Saintpaul	7	(5)	0		5	(7)	2	(40)
Brandenburg	2	(1)	0		5	(7)	0	
Thompson	4	(3)	1	(25)	4	(5)	0	
Typhimurium	1	(1)	1	(100)	4	(5)	2	(50)
Enteritidis	0	(0)	0		4	(5)	0	
Bareilly	7	(5)	0		4	(5)	0	
Infantis	6	(4)	4	(67)	4	(5)	3	(75)
Nagoya	0	(0)	0		3	(4)	0	
Newport	8	(5)	2	(25)	3	(4)	1	(33)
Blockley	3	(2)	3	(100)	2	(3)	2	(100)
Mikawashima	1	(1)	0		2	(3)	0	
Litchfield	1	(1)	0		2	(3)	1	(50)
Bovismorbificans	2	(1)	0		2	(3)	0	
Give	0	(0)	0		1	(1)	1	(100)
Havana	1	(1)	1	(100)	1	(1)	0	
Weltevreden	0	(0)	0		1	(1)	0	
Braenderup	5	(3)	1	(20)	1	(1)	0	
Manhattan	0	(0)	0		1	(1)	1	(100)
Schwarzengrund	17	(11)	16	(94)	1	(1)	1	(100)
Mbandaka	3	(2)	0		1	(1)	0	
Colindale	2	(1)	0		1	(1)	0	
Narashino	0	(0)	0		1	(1)	1	(100)
Senftenberg	0	(0)	0		1	(1)	0	
Willemstad	0	(0)	0		1	(1)	1	(100)
Untypable (O4:i:-)	0	(0)	0		6	(8)	6	(100)
Untypable (O8:-:1,5)	1	(1)	0		1	(1)	0	
Untypable (O8:e,h:-)	0	(0)	0		1	(1)	0	
Untypable (OUT:r:1,5)	0	(0)	0		1	(1)	1	(100)
Others	75	(48)	11	(15)	0		0	
Total	157		44	(28)	77		27	(35)

Highlighted serotypes show common isolations between sewage and humans. Total is not 100% due to the approximation of values.

**Table 3:** Isolation months and number of isolates of shared serotypes.

Serotype	Sources	2016							2017												2018												2019							Total
		6	7	8	9	10	11	12	1	2	3	4	5	6	7	8	9	10	11	12	1	2	3	4	5	6	7	8	9	10	11	12	1	2	3	4	5	6	7	
Agona	Sewage	-	-	1	-	-	-	-	-	1	1	1	-	1	-	-	1	-	-	-	1	-	-	-	-	-	-	-	-	-	-	-	-	-	-	-	-	-	-	7
	Human	1	-	-	-	-	-	-	-	-	-	-	1	-	1	-	1	-	-	-	-	-	-	-	-	-	-	-	-	-	-	-	-	-	1	-	-	1	1	7
Bareilly	Sewage	-	-	-	-	1	-	-	-	-	-	-	-	-	-	-	1	-	-	-	-	-	-	1	-	-	1	-	1	1	-	-	-	1	-	-	-	-	-	7
	Human	-	-	-	-	-	-	-	-	-	-	-	-	-	-	-	-	-	1	-	1	-	-	-	-	-	1	-	-	-	-	-	-	-	-	-	-	1	-	4
Bovismorbificans	Sewage	-	-	-	-	-	-	-	-	-	-	-	-	-	-	-	-	-	-	-	-	-	-	-	-	-	-	-	-	-	-	-	-	-	-	-	-	2	-	2
	Human	-	-	-	-	-	-	-	-	-	-	-	-	-	-	-	-	-	-	-	-	-	-	-	-	-	-	-	-	-	-	-	-	-	-	-	-	2	-	2
Brandenburg	Sewage	-	-	-	-	-	-	-	-	-	-	-	-	-	-	-	-	-	-	-	-	-	-	-	-	-	-	-	-	-	1	-	-	-	-	1	-	-	-	2
	Human	-	-	1	-	-	-	-	-	-	-	-	-	-	-	-	-	-	-	-	-	-	-	-	-	-	-	-	-	-	1	1	-	-	-	-	-	1	-	4
Colindale	Sewage	-	-	-	-	-	-	-	-	-	-	-	-	-	-	-	-	-	-	-	-	-	-	-	-	-	-	2	-	-	-	-	-	-	-	-	-	-	-	2
	Human	-	-	-	-	-	-	-	-	-	-	-	-	-	-	-	-	-	-	-	-	-	-	-	-	-	-	1	-	-	-	-	-	-	-	-	-	-	-	1
Infantis	Sewage	-	-	-	-	-	-	-	-	-	-	-	-	-	1	-	-	1	1	-	-	-	-	-	-	-	1	-	-	-	1	-	1	-	-	-	-	-	-	6
	Human	-	-	-	-	-	-	-	-	-	-	-	-	-	-	-	-	-	1	-	-	-	-	-	-	-	-	-	-	-	-	-	-	-	1	-	-	-	1	3
Litchfield	Sewage	-	-	-	1	-	-	-	-	-	-	-	-	-	-	-	-	-	-	-	-	-	-	-	-	-	-	-	-	-	-	-	-	-	-	-	-	-	-	1
	Human	-	-	-	1	-	-	-	-	-	-	-	-	-	-	-	-	-	-	-	-	-	-	-	-	-	-	-	-	1	-	-	-	-	-	-	-	-	-	2
Mbandaka	Sewage	-	-	-	-	-	-	-	-	-	-	-	-	-	-	-	-	-	-	-	-	1	-	-	1	-	-	-	-	-	-	-	-	-	1	-	-	-	-	3
	Human	-	-	-	-	-	-	-	-	-	-	-	-	-	-	-	-	-	-	-	-	-	-	-	-	1	-	-	-	-	-	-	-	-	-	-	-	-	-	1
Newport	Sewage	-	-	1	-	-	-	-	1	-	-	-	-	-	-	-	-	-	-	-	-	-	-	-	2	2	-	-	-	1	-	-	-	-	-	-	1	-	-	8
	Human	-	-	-	1	-	-	-	-	-	-	-	-	-	-	-	-	-	-	-	-	-	1	-	-	-	-	-	1	-	-	-	-	-	-	-	-	-	-	3
Saintpaul	Sewage	-	-	-	-	-	1	-	1	-	1	-	-	1	-	1	-	1	-	1	-	-	-	-	-	-	-	-	-	-	-	-	-	-	-	-	-	-	-	7
	Human	-	-	2	-	-	-	-	-	-	-	-	-	-	-	2	-	-	-	-	-	-	-	-	-	-	-	-	-	1	-	-	-	-	-	-	-	-	-	5
Schwarzengrund	Sewage	-	1	-	1	-	-	1	1	-	1	2	1	-	1	1	-	-	-	-	-	-	-	-	-	-	1	-	1	-	1	1	1	-	-	1	-	1	-	17
	Human	-	-	-	-	-	-	-	-	-	-	-	1	-	-	-	-	-	-	-	-	-	-	-	-	-	-	-	-	-	-	-	-	-	-	-	-	-	-	1
Stanley	Sewage	-	-	-	1	-	-	-	1	-	1	-	-	-	-	1	-	-	-	-	-	-	-	-	-	-	-	-	-	-	-	-	-	-	-	-	-	-	-	4
	Human	-	-	-	-	-	-	-	-	-	1	-	-	-	-	1	2	-	-	-	-	-	-	-	-	1	-	-	-	-	-	-	-	-	-	-	-	-	1	6
Untypable (O8:-:1,5)	Sewage	-	-	-	-	-	-	-	-	-	-	-	-	-	-	1	-	-	-	-	-	-	-	-	-	-	-	-	-	-	-	-	-	-	-	-	-	-	-	1
	Human	-	-	-	-	-	-	-	-	-	-	-	-	-	1	-	-	-	-	-	-	-	-	-	-	-	-	-	-	-	-	-	-	-	-	-	-	-	-	1

Highlighted numbers show the isolates within a 1-month period from each other.
